# Correlation of Somatostatin Receptor 2 Expression, 68Ga-DOTATATE PET Scan and Octreotide Treatment in Thymic Epithelial Tumors

**DOI:** 10.3389/fonc.2022.823667

**Published:** 2022-02-07

**Authors:** Anja C. Roden, Sagar Rakshit, Geoffrey B. Johnson, Sarah M. Jenkins, Aaron S. Mansfield

**Affiliations:** ^1^ Department of Laboratory Medicine and Pathology, Mayo Clinic, Rochester, MN, United States; ^2^ Division of Medical Oncology, Mayo Clinic, Rochester, MN, United States; ^3^ Department of Radiology, Mayo Clinic, Rochester, MN, United States; ^4^ Department of Immunology, Mayo Clinic, Rochester, MN, United States; ^5^ Division of Clinical Trials and Biostatistics, Mayo Clinic, Rochester, MN, United States

**Keywords:** somatostatin receptor 2, SSTR2, DOTATATE scan, octreotide treatment, thymoma, thymic carcinoma, atypical carcinoid tumor, thymic neuroendocrine tumor

## Abstract

Somatostatin receptor 2 (SSTR2) has been shown to be expressed in a subset of neuroendocrine tumors and carcinomas and plays a role in imaging studies and guiding therapy. Patients with tumors expressing SSTR2 may be successfully treated with somatostatin inhibitors or radiolabeled somatostatin analogues. We studied SSTR2 expression in TET and correlated it with 68Ga-DOTATATE PET/CT or 68Ga-DOTATATE PET/MR results and treatment outcome. An institutional database of TET was searched for thymoma, thymic carcinoma, and thymic neuroendocrine tumor (TNET) with available resection specimens. Cases were subtyped (2021 WHO classification) and staged (8^th^ AJCC/UICC staging). A section was stained with anti-SSTR2 antibody (clone UMB1). Percent tumor cells with membranous staining was recorded if present in ≥1% of tumor cells. Medical records were searched for 68Ga-DOTATATE PET scans and treatment. Statistical analysis was performed. Eighty patients (1969-2021) with a median age of 61.3 years (range, 19.1-87.3) (37 males, 46.3%) had thymic carcinoma (N=33), TNET (N=7), or thymoma (N=40). SSTR2 expression was identified in 29 (of 80, 36.3%) TET including 2/2 (100%) small cell carcinomas, 2/5 (40.0%) atypical carcinoid tumors, 16/23 (69.6%) squamous cell carcinomas, 2/2 (100%) lymphoepithelial carcinomas, 1/1 (100%) adenosquamous carcinoma, and 6/40 (15.0%) thymomas. SSTR2 expression in ≥50% of tumor cells (vs 1-49%) was associated with younger age (p=0.023) and shorter recurrence/metastasis-free survival (p=0.007). 68Ga-DOTATATE PET scans (N=9) revealed a Krenning score of 3 in patients with atypical carcinoid tumor, small cell carcinoma, and squamous cell carcinoma (N=1 each) with SSTR2 expression in 95, 100, and 5% of tumor cells, respectively. Scans with Krenning scores of ≤2 (N=5) were seen in tumors with no SSTR2 expression in 80% of cases and a single atypical carcinoid tumor with SSTR2 expression in 10% of tumor cells. One scan resulted as “increased uptake” was in a patient with no SSTR2 expression. In conclusion, 68Ga-DOTATATE PET scans correlated with SSTR2 expression in TET in most patients and appeared to be useful to identify patients with TET who may be amenable to treatment with somatostatin analogues. Larger studies including more patients with 68Ga-DOTATATE PET scans are necessary to independently and prospectively validate our findings.

## Introduction

Thymic epithelial tumors (TET) including thymomas, thymic carcinomas, and thymic neuroendocrine tumors (TNET) are malignant neoplasms. Although these tumors are rare, they together represent the most common solitary lesions in the prevascular mediastinum (1). Specifically thymic carcinomas and TNETs are often diagnosed at high stage and their large size and infiltration of vital organs commonly prevents complete resection. For instance, 71 to 79% of patients with thymic carcinoma present at stages III or IV and complete resection is reported in only 46 to 69% of patients with 5-year overall survival rates of 52 to 64% and disease-free survival of 41% (2–6). In TNET, the outcome depends on the histologic subtype with 5-year overall survivals of 50 to 70% for typical and atypical carcinoid tumors, 30 to 66% for large cell neuroendocrine carcinomas, and 0% with a median survival of 13-26 months for small cell carcinomas (7).

Predictive biomarkers are scarce in TET and therefore options for targetable therapy are currently very limited. Thymomas and thymic carcinomas have very low tumor mutational burdens with 0.48 mutations and 1.2 mutations/Mb, respectively with a rare thymic carcinoma reported to harbor 21.3 mutations/Mb (8–10). *GTF2I* mutation is the most common molecular alteration in TET, specifically in type A and AB thymomas where it occurs in 70 to 100% of cases. In contrast to thymomas, *GTF2I* mutation is only identified in 0 to 8% of thymic carcinomas, and has not been described in TNETs (8, 10, 11). Moreover, no therapies are currently available to target *GTF2I*. *KIT* mutations occur in 6 to 20% of thymic carcinomas although only a few tumors harbor an activating *KIT* mutation and therefore only some patients may benefit from receptor tyrosine kinase inhibitors (12–14). Other mutations in thymic carcinomas include *TP53*, *CDKN2A*, *cyclin D1*, *FGFR3*, and *ALK* among others, some of which may potentially be targetable (8, 12, 13, 15, 16). While PD-L1 expression has been identified in 27 to 80% of thymic carcinomas and 23 to 54% of thymomas, given the unique immune status of the thymic gland, immune checkpoint inhibitor treatment requires further investigation (9, 17–20). Taken together, predictive biomarkers are still strongly sought to predict tumor responsiveness of TET to targeted therapy.

Somatostatin receptor 2 (SSTR2) is a G protein-coupled cell surface receptor in the family of somatostatin receptors that has multiple roles *via* adenylate cyclase, calcium influx, and has effects on cell cycling angiogenesis, apoptosis, and growth factor signalling (21, 22). The activation of the receptor by extracellular ligands has been shown to lead to inhibition of cell proliferation (23). In contrast, in a study of small cell lung carcinomas in which 48% of tumors expressed SSTR2, high SSTR2 expression (defined in that study as SSTR2 expression in tumor tissue 1+ or greater or immunohistochemical score ≥ 1) was associated with worse 2-year survival when compared to low expression of SSTR2 (21). These results were largely attributed to limited stage disease and not seen in patients with extensive stage disease. The authors concluded that SSTR2 signaling in small cell lung cancer may support tumor growth and maintenance. Indeed, that study further showed that downregulation of SSTR2 leads to increased apoptosis and decreased tumor growth in small cell lung carcinoma (21).

SSTR2 has also been recognized as an imaging and treatment target in various neoplasms including neuroendocrine tumors such as paragangliomas and small cell lung carcinomas, but also other malignancies such as thyroid carcinomas, and EBV-driven and non-EBV-driven nasopharyngeal carcinomas (22, 24–26). There are two primary mechanisms by which treatment may be delivered including 1) SSTR2 analogues that rely on SSTR2 signaling-induced changes and 2) the use of SSTR2 as a targeting molecule to deliver a cytotoxic or radioactive payload in form of an antibody-drug conjugate (22). For instance, DOTATATE and DOTA-Tyr-octreotide (DOTA-TOC) are clinically available somatostatin analogues that bind to SSTR2 (26). Patients with tumors expressing SSTR2 have been shown to be successfully treated with peptide somatostatin inhibitors such as octreotide or DOTATATE (27). These agents can be linked to radionuclides like 68-gallium (68Ga) and 177-lutetium (177Lu) for both imaging and therapeutic applications respectively. For instance, Thakur et al. found that SSTR2 is higher expressed in thyroid carcinomas and medullary carcinomas than in normal thyroid tissue and most patients with metastatic thyroid cancer showed positive 68Ga-DOTATATE uptake indicating that SSTR2 is expressed by these tumors (28). Treatment of mice with 177Lu DOTATATE resulted in tumor growth reduction (28). Furthermore, in a randomized, placebo-controlled study of the somatostatin analogue lanreotide in patients with SSTR-positive (by scintigraphy) grade 1 or 2 neuroendocrine tumors with a Ki-67 proliferative index of <10% originating in the pancreas, midgut, or hindgut or of unknown origin lanreotide treatment was associated with significantly prolonged progression-free survival of 65% at 24 months when compared to 33% for the placebo group (29).

Although DOTATATE PET scans are part of the clinical workup of some TET, specifically TNETs and octreotide treatment has been used in a subset of TET including thymomas, thymic carcinomas, and TNETs, SSTR2 expression has only been studied in small case series or case reports (30–32). Furthermore, the correlation between SSTR2 expression and results of 68Ga-DOTATATE PET scans in TET is largely unknown. Therefore, we studied the expression of SSTR2 in TET and its correlation with results of DOTATATE PET scans and treatment outcome.

## Methods

### Patients

In this retrospective study, an institutional database of TET (1941-2021) was searched for thymoma, thymic carcinoma, and TNET from patients who underwent surgery. All cases were reviewed by a thoracic pathologist (ACR) to confirm the diagnosis. TETs were subtyped according to the 2021 WHO classification (33) and staged using the 8^th^ AJCC/UICC staging manual (34). To avoid possible misinterpretation of SSTR2-expression on tumor cells due to potentially high numbers of SSTR2-expressing thymocytes only type A and B3 thymomas, micronodular thymomas with lymphoid stroma, thymic carcinomas, and TNETs were included in the study. Patient demographics and outcomes were recorded from medical records. Medical records were also searched for 68Ga-DOTATATE PET scans and treatment. The study was approved by the Mayo Clinic Rochester Institutional Review Board (#10-003525).

### Immunohistochemistry

Formalin-fixed paraffin-embedded tissue blocks were cut at 4μm and stained with anti-SSTR2 antibody (clone UMB1, Abcam, Boston, MA). Percent tumor cells with membranous staining was recorded if present in ≥ 1% of tumor cells. The staining evaluation was modified from the evaluation used by Volante et al. (35) Similar to the evaluation system by Volante et al. no expression or focal or diffuse cytoplasmic expression of SSTR2 were regarded as negative. Membranous SSTR2 staining in 1 to 49% of tumor cells and 50 to 100% of tumor cells were considered positive. Staining intensity was noted as negative, weak, moderate, or strong.

### 68Ga-DOTATATE PET Scans

Available 68Ga-DOTATATE PET/CT and PET/MR scans were reviewed by a nuclear medicine physician and radiologist (GBJ) and a Krenning score was applied. Briefly, Krenning score is a qualitative measure of relative uptake of 68Ga-DOTATATE in tumors versus the physiologic uptake in the internal organs of the liver and spleen, with a Krenning score of 1 being negative and tumor activity far below liver activity, a Krenning score of 2 being mildly positive with activity slightly less than liver, a Krenning score of 3 being positive with activity higher than liver, and a Krenning score of 4 being very positive with activity above spleen. Scans were performed on GE Discovery 710, GE Discovery MI and Siemens Vision 600 PET/CT or GE Signa PET/MR scanners. Uptake was 60 minutes plus or minus 5 minutes in all patients, and the injected dose was 5.2 mCi 68Ga-DOTATATE IV +/- 10% IV.

### Statistical Analysis

Continuous and ordinal characteristics were compared between SSTR2 expression groups with Wilcoxon rank-sum tests, and categorical characteristics were compared with Fisher’s exact tests. The WHO type was compared using the following compressed categories: thymoma, thymic carcinoma, and thymic neuroendocrine tumors (including small cell carcinomas and atypical carcinoid tumors). Recurrence/metastasis-free survival (RFS) and overall survival (OS) were compared between groups with likelihood ratio tests from Cox proportional-hazards regression models. Five-year RFS and OS were summarized using the Kaplan-Meier method along with 95% confidence intervals (CI). P-values less than 0.05 were considered statistically significant. All analyses were performed using SAS version 9.4 (SAS Institute Inc., Cary, NC).

## Results

### Patient Characteristics and Tumor Histology

Eighty patients surgically treated between 1969 and 2021 were included in the study. Demographics of the study population are summarized in **Table 1**. These 80 patients carried thymomas (N=40, 50%), thymic carcinomas (N=33, 41.3%), and TNETs (N=7, 8.8%). Tumor histology is detailed in **Table 2**.

**Table 1:** Demographics, treatment, and outcome of study population.

Feature	Results
Study population, N	80
Male sex, N (%)	37 (46.3)
Age, years, median (range)	61.3 (19.1-87.3)
Surgical treatment, N (%)	
Complete resection	68 (85.0)
Incomplete resection	7 (8.8)
Biopsy	3 (3.8)
Recurrence/metastasis^a^	2 (2.5)
Tumor size in cm, median (range)^b^	5.0 (0.5-23.0)
pT stage, N (%)^c^	
T1a	50 (66.7)
T2	1 (1.3)
T3	23 (30.7)
T4	1 (1.3)
pN stage ^c^	
N0	36 (72.0)
N1	13 (26.0)
N2	1 (2.0)
NX	25
pM stage ^c^	
M0	68 (90.7)
M1a	3 (4.0)
M1b	4 (5.3)
TNM Stage	
I	28 (52.8)
II	1 (1.9)
IIIA	4 (7.5)
IVA	15 (28.3)
IVB	5 (9.4)
N/A	27
Additional Therapies, N (%)	34 (42.5)
Octreotide^d^	3
Adjuvant radiation	10
Adjuvant chemoradiation	9
Neoadjuvant & adjuvant chemotherapy & adjuvant radiation	3
Adjuvant chemotherapy	2
Neoadjuvant chemotherapy	2
Neoadjuvant radiation	2
Neoadjuvant chemotherapy	2
Neoadjuvant chemotherapy & adjuvant radiation	2
Neoadjuvant chemoradiation	1
Neoadjuvant & adjuvant radiation	1
Unknown	3
Outcome	
Follow up available, N	78
Follow up time in years, median (range)	3.1 (0.1-19.6)
Metastasis and/or recurrence, N (5-year estimate, %)^c,e^	23 (41.3 [95% CI: 27.4%-55.2%])
Ongoing disease after incomplete resection, N	1
Alive, N (5-year estimate, %)	53 (68.1 [95%CI: 55.5%-80.7%])
Alive without disease, N	43
Alive with disease, N	10
Death, N (5-year estimate, %)	25 (32.9 [95% CI: 19.3%-45.5%])
Died due to disease, N	10
Died due to other cause, N	5
Died due to unknown cause, N	10

a In these 2 patients the specimen at time of recurrence/metastasis was tested for SSTR2 expression; date of resection of primary tumor was used for outcome analysis;

b Only including completely resected primary tumors;

c Only including resected tumors;

d All 3 patients received octreotide in addition to other additional treatment;

e Data available in 75 patients; N/A, not available.

**Table 2:** Histopathologic features and expression of SSTR2 in thymic epithelial tumors.

Tumor Histology	Number of Cases (%)	Number of SSTR2-negative Cases (%)	Number of Total Cases Expressing SSTR2 in 1-100% of Tumor Cells (%) (median % SSTR-positive tumor cells, range)	Number of Cases Expressing SSTR2 in 1-49% of Tumor Cells (%) (median % SSTR-positive tumor cells, range)	Number of Cases Expressing SSTR2 in ≥ 50% of Tumor Cells (%) (median % SSTR-positive tumor cells, range)	P-Value [Pos vs Neg^a^, 1-49% vs ≥50%^b^]
Total number of cases, N (%)	80	51 (63.7)	29 (36.3)	17 (21.3)	12 (15.0)	
Age in years, median (range)	61.3 (19.1-87.3)	63.9 (28.1-83.9)	56.3 (19.1-87.3)	61.6 (29.7-87.3)	49.4 (19.1-65.6)	0.09, 0.023
Thymoma	40 (50.0)	34 (85)	6 (15.0) (1.5; 1-5)	6 (15.0) (1.5; 1-5)	0	<0.001, 0.07
Type A	24	21 (87.5)	3 (12.5) (1;1-5)	3 (12.5) (1;1-5)	0	
Type B3	9	8 (88.9)	1 (11.1) (1)	1 (11.1) (1)	0	
Micronodular thymoma with lymphoid stroma	7	5 (71.4)	2 (28.6) (3.5,2-5)	2 (28.6) (3.5,2-5)	0	
Thymic carcinoma	33 (41.3)	14 (42.4)	19 (57.6)	9 (27.3)	10 (30.3)	
			(50; 1-100)	(10; 1-30)	(85; 50-100)	
Squamous cell carcinoma	23	7 (30.4)	16 (69.6) (40;1-100)	8 (34.8) (10;1-30)	8 (34.8) (90;50-100)	
Adenocarcinoma	4	4 (100.0)	0	0	0	
Mucoepidermoid carcinoma	2	2 (100.0)	0	0	0	
Lymphoepithelial carcinoma	2	0 (0.0)	2 (100.0)	0	2 (100.0)	
			(70; 70 each)		(70; 70 each)	
Adenosquamous carcinoma	1	0 (0.0)	1 (100.0) (5)	1 (100.0) (5)	0	
Undifferentiated carcinoma	1	1 (100.0)	0	0	0	
Thymic neuroendocrine tumor	7 (8.8)	3 (42.9)	4 (57.1)	2 (28.6)	2 (28.6)	
			(52.5; 2-100)	(6.0; 2-10)	(97.5; 95-100)	
Atypical carcinoid tumor	5	3 (60.0)	2 (40.0) (52.5;10-95)	1 (20.0) (10)	1 (20.0) (95)	
Small cell carcinoma	2	0 (0.0)	2 (100.0) (51;2-100)	1 (50.0) (2)	1 (50.0) (100)	
TNM Stage						0.10, 0.22
I	28 (52.8)	20 (71.4)	8 (28.6) (3.5; 1-70)	7 (25.0) (2; 1-10)	1 (3.6) (70)	
II	1 (1.9)	1 (100.0)	0	0	0	
IIIA	4 (7.5)	1 (25.0)	3 (75.0) (90; 10-95)	1 (25.0) (10)	2 (50.0) (92.5; 90-95)	
IVA	15 (28.3)	8 (53.3)	7 (46.7)	3 (20.0)	4 (26.7)	
			(90.0; 1-100)	(10.0; 1-30)	(95.0; 90-100)	
IVB	5 (9.4)	2 (40.0)	3 (60.0) (5.0; 5-70)	2 (40.0) (5 each)	1 (20.0) (70)	
N/A	27	19 (70.4)	8 (29.6)	4 (14.8)	4 (14.8)	
			(37.5; 1-100)	(7.5; 1-25)	(75.0; 50-100)	

a P-value for comparison of positive vs negative SSTR2 expression (1-100% vs <1%) between thymoma, thymic carcinoma, and thymic neuroendocrine tumor.

b P-value for comparison of SSTR2 expression (1-49% vs ≥50%) between thymoma, thymic carcinoma and thymic neuroendocrine tumor (excluding those with <1% expression). N/A, not available.

The median age of the study population was 61.3 years with a slight female predominance (53.7%). Patients with thymoma were significantly older at time of surgery than patients with thymic carcinoma (median age [range], 64.6 years [28.1-87.3] vs 52.8 [19.1-79.6], respectively) (p=0.02) and patients with TNET (median age [range], 64.6 years [28.1-87.3] vs 44.1 [29.7-61.4], respectively) (p=0.004). There was no difference in age between patients with thymic carcinoma and patients with TNET (p=0.11). Most patients (85%) underwent complete resection. The median tumor size of resected tumors was 5.0 cm. Most tumors (52.8%) were of stage I. Additional neoadjuvant and/or adjuvant therapy was given in 42.9% of patients; 3 patients also received octreotide.

Follow up was available in 78 patients for a median of 3.1 years (range, 0.1-19.6). The outcome of patients is detailed in **Table 1**. Twenty-three patients had a metastasis and/or recurrence within 0.2 to 4.5 years after resection of the primary tumor (5-year rate of metastasis/recurrence, 41.3%, 95% CI: 27.4%-55.2%). Most patients (43 of 78) were alive without disease at the time of last follow-up. Ten (of 78) died due to TET. All patients who died due to disease had either thymic carcinoma or TNET. The 5-year overall survival was 68.1% (95% CI: 55.5%-80.7%).

### SSTR2 Expression of Tumors

SSTR2 expression (1-100%) was identified in 29 (of 80, 36.3%) TET. Results of SSTR2 expression are detailed in **Table 2**. SSTR2 expression was more common among thymic carcinomas (57.6%) and TNET (57.1%) as compared to thymomas (15.0%, p<0.001) (**Table 2**). Furthermore, among those expressing SSTR2, there was a trend of higher expression of SSTR2 (≥ 50%) in thymic carcinomas (10 of 19, 52.6%) and TNETs (2 of 4, 50%) than in thymomas (0 of 6) (p=0.07) (**Table 2**).

SSTR2 expression was strong in 19 (of 29, 65.5%), moderate in 7 (24.1%), and weak in 3 (10.3%) TET. Weak expression was seen in 2 thymomas with 1% of tumor cells expressing SSTR2 and in an adenosquamous carcinoma with 5% of tumor cells expressing SSTR2.

As compared to those with lower SSTR2 expression (1-49% of tumor cells), those with ≥ 50% tumor cells expression were younger in age (p=0.023). Also, SSTR2 expression in ≥ 50% of tumor cells trended to be more common in thymic carcinomas when compared to TNET and thymoma (p=0.056). SSTR2 expression (1-100%) was more commonly seen in TET of patients with high stage (stages IIIA-IVB; 13 of 24, 54.2%) compared to patients with low stage (stage I; 8 of 28, 28.6%) tumors although that was not statistically significant (p=0.10). The distribution of tumor stage in relationship to the subtype of TET is summarized in **Table 3**.

**Table 3:** Stage distribution of thymic epithelial tumors of the study population (N=53)^a^.

Stage	Thymoma Type (N)	Thymic Carcinoma	Thymic Neuroendocrine Tumor
I	A (16)	Squamous cell carcinoma (2)	Small cell carcinoma (1)
	B3 (5)	Adenocarcinoma (1)	
	Micronodular thymoma with lymphoid stroma (3)		
II		Squamous cell carcinoma (1)	
IIIA	Micronodular thymoma with lymphoid stroma (1)	Squamous cell carcinoma (3)	
IVA	B3 (1)	Squamous cell carcinoma (8)	Atypical carcinoid tumor (4)
		Adenocarcinoma (2)	
IVB		Squamous cell carcinoma (1)	Atypical carcinoid tumor (1)
		Adenosquamous carcinoma (1)	
		Lymphoepithelial carcinoma (1)	
		Undifferentiated carcinoma (1)	

a Only includes patients with primary tumors and available staging.

SSTR2 expression in ≥ 50% of tumor cells was associated with worse RFS with an estimated 5-year RFS of 11.4% (95% CI: 0%-32.5%) vs 72.7% (95% CI: 46.4%-99.0%) in patients with 1-49% of tumor cells expressing SSTR2 (p=0.007). The estimated 5-year RFS among patients with <1% SSTR2 expression was 67.8% (95% CI: 51.3%-84.3%; p=0.10 for <1% vs 1-100% SSTR2 expression). SSTR2 expression was not associated with 5-year OS (estimated 66.3% [95% CI: 34.4%-98.2%], 67.9% [95% CI: 41.7%-94.1%], 69.2% [53.5%-84.9%] for patients with ≥ 50%, 1-49%, <1% tumor cells expressing SSTR2, respectively; p=0.71 for <1% vs 1-100% SSTR2 expression; p=0.96 for ≥ 50% vs 1-49% SSTR2 expression).

### Results of 68Ga-DOTATATE Scans and Correlation With Clinicopathologic Features and SSTR2 Expression

**Figure 1:**
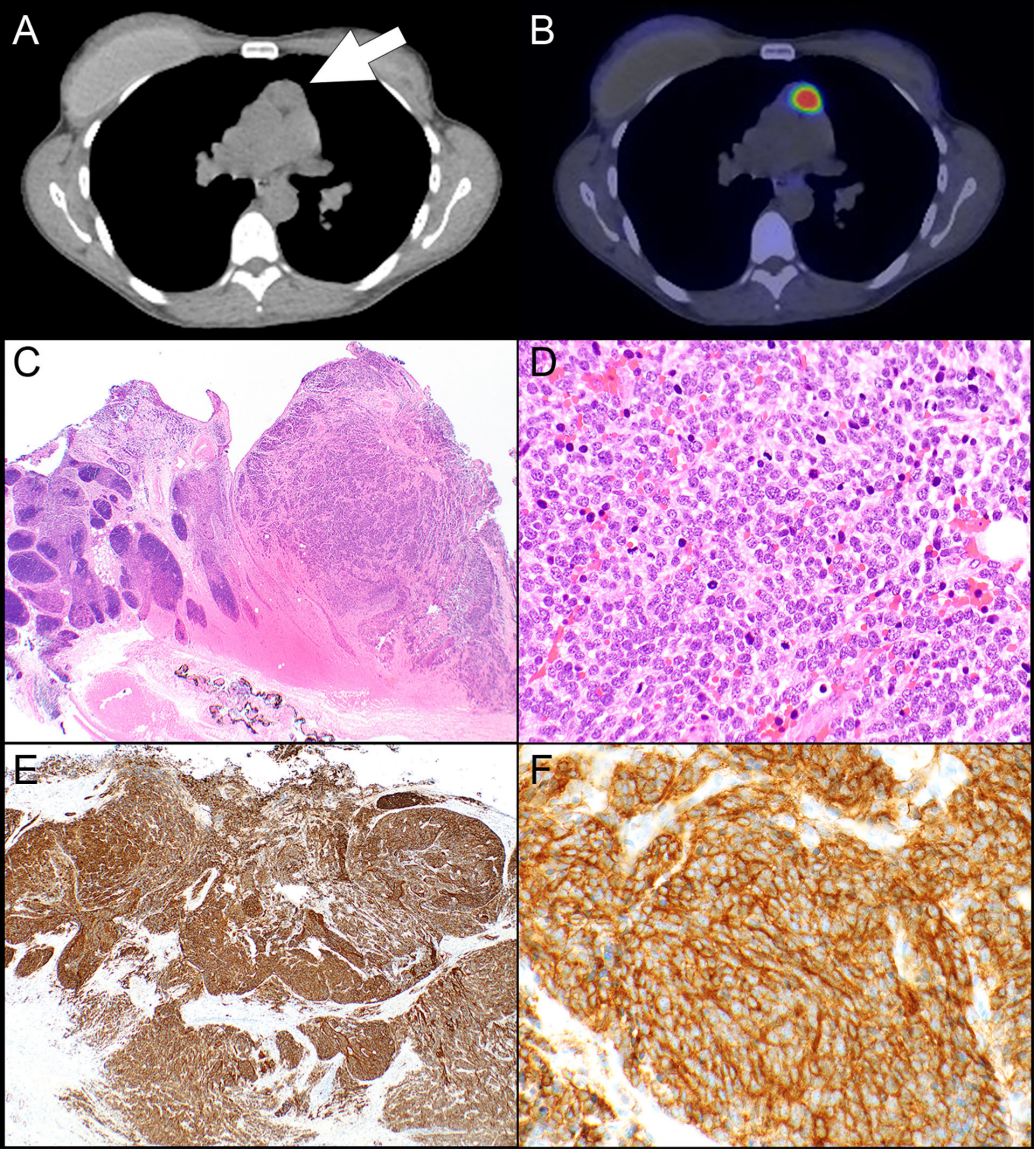
Patient #8. **(A)** A computed tomography scan shows a prevascular mediastinal lesion (arrow) that takes up 68Ga on a DOTATATE PET scan which was interpreted as Krenning score 3 **(B)**. **(C)** The resection specimen shows sheets and nests of neoplastic cells (right side) in a background of thymic gland (left side). **(D)** The neoplastic cells are round with high nuclear-to-cytoplasmic ratio and high mitotic activity. The neoplastic cells are positive for keratin CAM5.2 (focal), synaptophysin, chromogranin (focal), and TTF-1 and negative for p63; Ki-67 shows a high proliferative index (stains not shown). **(E)** SSTR2 expression is diffuse and strong in virtually all tumor cells in a membranous and cytoplasmic expression pattern **(F)**. Magnification, H&E x 20 **(C)**, x 400 **(D)**, SSTR2 x 40 **(E)**, x 400 **(F)**.

**Figure 2:**
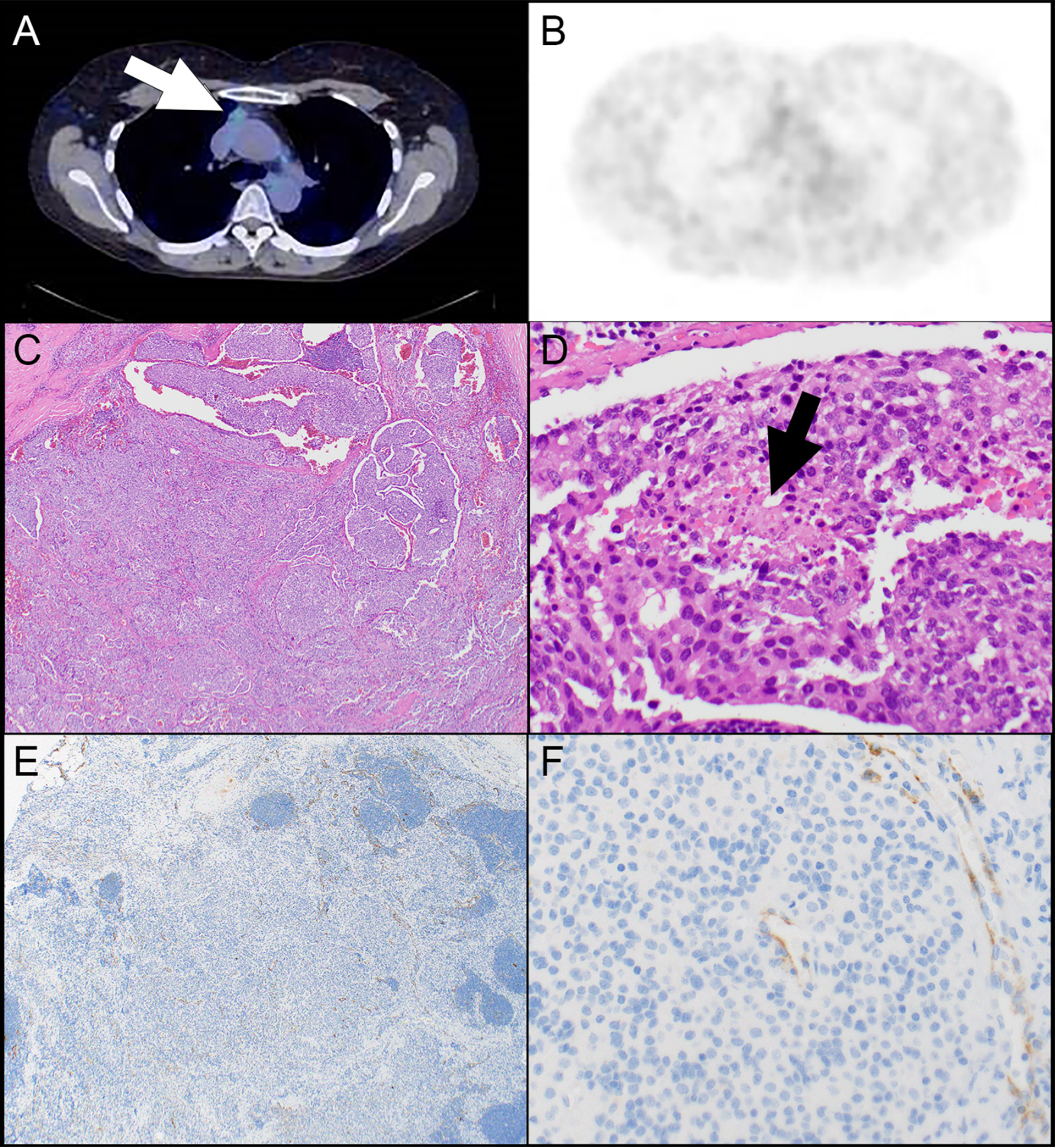
Patient #4. **(A)** A computed tomography scan shows a prevascular mediastinal lesion (arrow) that shows 68Ga take up on a DOTATATE PET scan that is much lower than liver (interpreted as Krenning score 1) **(B)**. **(C)** A nested neoplasm is comprised of round to oval cells with a small amount of cytoplasm and associated with focal necrosis (**D**, arrow). **(E)** There is no expression of SSTR2 in tumor cells. **(F)** Note SSTR2 expression in endothelial cells serving as internal control. Magnification, H&E x 40 **(C)**, x 400 **(D)**, SSTR2 x 40 **(E)**, x 400 **(F)**.

68Ga-DOTATATE PET scans were available in 9 patients. The results of the scans are presented in **Table 4**. Scans were available from 6 patients with TNET including 5 patients with atypical carcinoid tumor and 1 patient with small cell carcinoma. In addition, scans were available from 2 patients with squamous cell carcinoma and one patient with type B3 thymoma. In 3 patients the scan was performed at some time after resection of the primary tumor. Krenning score 3 was identified in cases of atypical carcinoid tumor, small cell carcinoma, and squamous cell carcinoma (N=1, each). The small cell carcinoma also showed strong expression of SSTR2 in 100% of the tumor cells (**Figure 1**), the atypical carcinoid tumor showed moderate SSTR2 expression in 95% of tumor cells and the squamous cell carcinoma exhibited strong SSTR2 expression in 5% of tumor cells. “Increased uptake” was reported in an additional squamous cell carcinoma which had 0% of tumor cell staining with SSTR2. However, that scan was not available for re-review. Krenning score 2 was identified in 2 atypical carcinoid tumors, score 1 in an atypical carcinoid tumor (**Figure 2**), and no uptake was seen in an atypical carcinoid tumor and the type B3 thymoma. Interestingly, while all tumors with scans showing Krenning scores 1 or 2 or no uptake had no expression of SSTR2, the atypical carcinoid tumor with no uptake on 68Ga-DOTATATE PET scan did show strong SSTR2 expression in 10% of the tumor cells. However, that scan was also not available for review.

**Table 4:** Summary of patients with available DOTATATE scan.

Case	WHO Type	pTNM	% (Intensity) SSTR2-positive Tumor Cells	Krenning Score/Description of Scan	Treatment with Octreotide/somatostatin analogue	Treatment & Outcome
1	Squamous cell carcinoma	T3N1M0	0	Increased uptake	No	Incomplete resection, adjuvant radiation
						No recurrent disease
						Died 4 yrs after resection of unknown cause
2	Atypical carcinoid tumor	T3N1M0	10 (moderate)	No uptake	No	Complete resection of 3.2 cm tumor; adjuvant radiation
						First metastasis at 15 months after resection
						Metastases/recurrence to lymph nodes, bone, pleura, mediastinum, lung, brain
						DOD 3.5 years after resection
3	Atypical carcinoid tumor	T3N1M0	95 (moderate)	3	Yes	MEN1 syndrome
						Neoadjuvant chemotherapy
						Complete resection of 12.5 cm tumor, adjuvant radiation, octreotide acetate (Sandostatin)
						First metastasis 3.3 years after resection
						Metastasis to pleura
						AWD 11.2 years after resection
4	Atypical carcinoid tumor (**Figure 2**)	T3N1M0	0	1	No	Complete resection of 4.4 cm tumor; adjuvant radiation
						First metastases/recurrence 3.2 years after resection
						Metastases to hilar lymph nodes, lung, mediastinum
						AWD 5.2 years after resection
5	Atypical carcinoid tumor	T1N1M0	0	2	No	Complete resection of 3.7 cm tumor; adjuvant chemotherapy and steroids
						First metastasis 0.3 years after resection
						Metastasis to breast
						AWD 2.8 years after resection
6	WHO type B3 thymoma, recurrent	N/A	0	Performed 4 months after resection Negative	No	Resection of recurrence 6 years after initial incomplete resection, adjuvant chemoradiation
						Alive without disease 11.7 years after resection of primary tumor.
7	Squamous cell carcinoma	T3NXM1b	5 (strong)	Performed at time of recurrence/metastasis 3	Yes	Neoadjuvant chemotherapy
						Complete resection of 5 cm tumor; metastases to lung and liver at time of initial diagnosis
						First metastasis at 1.4 years after resection
						Metastasis to lung
						4.5 years after resection metastases to lymph nodes, pleura, pericardium, lung - at that time Ga68-DOTATATE scan; treatment with Octreotide for 3 months until diagnosed with breast carcinoma
						AWD 7.9 years after resection of primary tumor
8	Small cell carcinoma (**Figure 1**)	T1NXM0	100 (strong)	3 Multiple postoperative scans with scores 3 or 4	Yes	Complete resection of 2.7 cm tumor; adjuvant chemoradiation,
						First metastasis 1 year after resection
						Metastases to bone; treatment with octreotide (Sandostatin) for 3 months; follow up Ga68-DOTATATE showed progressive disease; chemoradiation
						AWD 1.6 years after resection of primary tumor
9	Atypical carcinoid tumor	T3N2M0	0	Multiple postoperative scans with score 2	No	Complete resection of 18.3 cm tumor; neoadjuvant & adjuvant chemotherapy & adjuvant radiation
						First metastasis/recurrence 0.8 years after resection
						Metastases to lung, pleura, mediastinal and abdominal lymph nodes
						AWD 0.9 years after resection of primary tumor

DOD, died of disease; AWD, alive with disease; N/A, not applicable.

All 3 patients with Krenning score 3 were treated with octreotide; none of the other patients received a somatostatin analogue treatment. One of these 3 patients who were treated with octreotide and had an atypical carcinoid tumor was metastasis and recurrence-free for 3.3 years after neoadjuvant chemotherapy, complete resection, adjuvant radiation, and adjuvant octreotide. Another patient was treated with octreotide when the thymic squamous cell carcinoma recurred. However, the patient was only treated for 3 months at which time a breast carcinoma was diagnosed and the treatment regimen was altered accordingly. Whether her disease progressed or regressed during those 3 months is unknown. The patient with small cell carcinoma was treated with octreotide for 3 months after bone metastases had developed; however, her disease progressed and therefore she underwent chemoradiation therapy 3 months later. All 3 patients were alive with disease at last follow up. None of the patients who did not undergo a 68Ga-DOTATATE PET scan were treated with octreotide.

Both lymphoepithelial carcinomas which were EBV-associated showed strong and diffuse expression of SSTR2. A poorly differentiated squamous cell carcinoma with marked tumor infiltrating lymphocytes, morphologically reminiscent of a lymphoepithelial carcinoma but without EBV association also showed expression of SSTR2 in 90% of tumor cells. These cases are detailed in **Table 4**. Results of DOTATATE PET scans are also detailed in **Table 4**.

## Discussion

Our study of 80 retrospectively collected TET including 33 thymic carcinomas and 7 TNETs revealed expression of SSTR2 in all thymic small cell carcinomas, lymphoepithelial carcinomas, and the single adenosquamous carcinoma, three-fourths of thymic squamous cell carcinomas, and 40% of atypical carcinoid tumors. Indeed, significantly more thymic carcinomas and TNETs expressed SSTR2 than thymomas. In slightly more than half of thymic carcinomas and TNETs SSTR2 was expressed in 50% or more tumor cells. In contrast, SSTR2 was only expressed in 15% of type A and B3 thymomas and micronodular thymomas with lymphoid stroma with expression in 5% or less of tumor cells. We also found that in all patients in whom a 68Ga-DOTATATE PET scan was available and the Krenning score was 3, the TET also expressed SSTR2 while in almost all cases of 68Ga-DOTATATE PET scan Krenning score 1 or 2 the tumor was negative for SSTR2 expression. Our results indicate that 68Ga-DOTATATE PET scan may serve as a screening tool for SSTR2 expression in TET although confirmation of SSTR2 expression in a resection specimen may reveal an occasional TET that is negative on 68Ga-DOTATATE PET scan. Our results suggest that at least a subset of thymic carcinomas and TNETs may be responsive to somatostatin analogue treatment or SSTR2 antibody-drug conjugates while type A and B3 thymomas and micronodular thymomas with lymphoid stroma are less likely to express SSTR2 and may not respond to such treatments.

Despite our findings of low SSTR2 expression in thymomas, literature has suggested that thymomas may show response to treatment with octreotide. For instance, in a study of 15 patients with unresectable or locally recurrent thymoma and 2 patients with thymic carcinoma at Masaoka stage III who had a positive octreotide scan and who were treated with octreotide and prednisone, a treatment response was found in 15 (88%) patients with a median reduction of tumor volume of 51% after 12 weeks of treatment; subsequent complete surgical resection was achieved in 9 (52%) patients (30). No response was observed in one of the carcinomas and a single AB thymoma. However, it is not clear whether the observed response in that study was indeed due to octreotide, a combined effect of prednisone and octreotide or a prednisone-only effect as studies have shown that prednisone by itself may lead to reduction in tumor volume of thymomas (36). In a similar study of 16 patients with advanced thymoma (N=10), thymic carcinoma (N=3), and thymic small cell carcinoma (N=3) unresponsive to conventional chemotherapy and positive octreotide scan, treatment with octreotide and prednisone resulted in a response rate of 37% with 1 complete response, 5 partial responses and 6 stable diseases (31). In a third clinical trial including patients with invasive, recurrent, or metastatic thymoma (N=32), thymic carcinoma (N=5), and thymic carcinoid tumor (N=1) not amenable to curative therapy and positive octreotide scan, all patients were treated with octreotide for 2 cycles (32). Patients with complete or partial response would continue to be treated with octreotide, patients with stable disease received prednisone and octreotide and patients with progressive disease were removed from the study. Two (5.3%) patients had complete response and 10 (25%) had partial response with an overall response rate of 30.3%. Fourteen (36.8%) patients had stable disease. Of 38 patients treated with octreotide alone, only 4 (10.5%) had a partial response. In 21 patients in whom prednisone was added there were 2 complete and 6 partial responses. All responses occurred in patients with thymoma. While these studies did not investigate the expression of SSTR2 in the tumor tissue, conceivably thymomas did express SSTR2 given the positive octreotide scans and the response to octreotide treatment in a small subset of patients. However, since tissue was not examined for SSTR2 expression in these thymomas it remains unclear whether SSTR2 was expressed in thymocytes, tumor cells or both. That may be one of the reasons for the discrepancy with our findings as we only included thymomas that were rich in neoplastic cells and either lacked or contained very few thymocytes. Furthermore, because none of the studies disclosed how many patients had undergone octreotide scan before patients were selected for these trials the incidence of SSTR2 expression in thymomas could not be estimated.

Both of our lymphoepithelial carcinomas, which were associated with EBV, showed strong and diffuse expression of SSTR2. This finding is consistent with a recent study by Lechner et al. that showed that in EBV-driven nasopharyngeal carcinomas the expression of SSTR2 is induced by EBV latent membrane protein 1 *via* the NF-κB pathway (23). Indeed in that study 252 of 311 (81%) nasopharyngeal carcinomas expressed SSTR2 and SSTR2 expression was enriched in EBV-positive and in non-keratinizing nasopharyngeal carcinomas. Similarly, Viswanathan et al. in a study of primary, recurrent, and/or undifferentiated nasopharyngeal carcinomas, showed multifocal to diffuse strong SSTR2 expression in 90% of tumors including 8 of 9 EBV-associated and one EBV-negative nasopharyngeal carcinoma (37). While one HPV-positive sinonasal carcinoma also showed patchy SSTR2 staining, the remaining HPV-positive sinonasal carcinomas, HPV-positive oropharyngeal squamous cell carcinomas, or oral cavity head and neck squamous cell carcinomas did not reveal any significant SSTR2 staining. Similarly, SSTR2 expression and 68Ga-DOTATATE uptake were observed in pulmonary lymphoepithelial carcinomas which also were associated with EBV (23, 38). However, not all carcinomas and TNETs that expressed SSTR2 in our and other studies were EBV-related and therefore other pathways for expression of SSTR2 must exist in these tumors. Interestingly, one of our study cases, a poorly differentiated thymic squamous cell carcinoma with marked lymphocytic tumor infiltrate at least suggestive of lymphoepithelial carcinoma but without EBV expression also showed strong and diffuse expression of SSTR2.

SSTR2 has been shown to be expressed in a subset of neuroendocrine tumors. A study by Popa et al. suggested that SSTR2 expression may be more common in low grade than in high grade neuroendocrine tumors (39). That study showed that 96% of G1, 71% of G2 and only 23% of G3 tumors expressed SSTR2. In addition, only 33% of neuroendocrine carcinomas were SSTR2 positive which was significantly lower than in well differentiated neuroendocrine tumors. We could not confirm that finding in TNETs as we found that only 40% of atypical carcinoid tumors expressed SSTR2 while 100% of small cell carcinomas expressed that marker. However, our overall number of TNETs was low which could have at least contributed to that discrepancy.

Evidence also suggests that SSTR2 expression may be more common in early than in late tumor stages in neuroendocrine tumors. In the study by Popa et al. of gastrointestinal neuroendocrine tumors, SSTR2 expression was 100% in tumors of early stage while only 56% of advanced stage tumors expressed SSTR2 (39). Interestingly, in our study SSTR2 expression was overall more commonly seen in advanced stage tumors (stages IIIA, IVA, IVB) than in stage I tumors although there was no statistically significant difference. However, that finding may have been biased by the case distribution as carcinomas and TNET were more commonly of high stage while thymomas were of low stage in our study. In addition, most of our cases were not neuroendocrine tumors in contrast to the study by Popa et al. Unfortunately, we were not able to compare the expression of SSTR2 between low and high stage for thymic carcinomas and TNETs separately due to the relative low number of cases.

In 9 of our cases 68Ga-DOTATATE PET scans were available. The results of the 68Ga-DOTATATE PET scans appeared to correlate with the expression of the SSTR2 protein by immunohistochemistry. Indeed in 2 patients with Krenning score of 3 tumors showed strong and diffuse SSTR2 expression. A third patient with a scan of Krenning score 3 had strong SSTR2 expression in 10% of tumor cells. In contrast, all cases with Krenning score 2 and lower had no SSTR2 expression in tumor cells except one atypical carcinoid tumor in which the scan reportedly did not show any uptake but the tissue showed moderate expression of SSTR2 in 10% of tumor cells. A potential reason for that apparent discrepancy may be that standard uptake value (SUV) per voxel is used to create a PET scan in 3D. SUV is a mathematical best estimate of how much radiotracer is in each voxel at a given point in time. In the case of a 68Ga-DOTATATE PET scan the point in time is 60 minutes after injection. SUV is not a direct measure of how many tumor cells express SSTR2. Nor is SUV a measure of how many copies of SSTR2 are expressed on a given cell. SUV is a measure of both and other factors as well. SUV is a measure of radiotracer per voxel, which is likely correlated to the amount of SSTR2 expressed on cells in a voxel. Therefore, if the tumor cells expressing SSTR2 are spread out in 3D space, perhaps due to dead cells, extracellular fibrosis or other structures in the voxel, the SUV will go down, and as such the Krenning score will go down. Our findings of the correlation between 68Ga-DOTATATE PET scan and SSTR2 expression in tissue are supported by the study by Lechner et al (23) of nasopharyngeal carcinomas. In that study the authors found a significant correlation between SSTR2 expression levels and uptake of 68Ga DOTATATE suggesting that this imaging modality may have potential as a noninvasive marker to monitor SSTR2 expression and as a target for SSTR2 receptor-targeted radionuclide therapy. This was also shown in an earlier study by Miederer et al. that evaluated a variety of neuroendocrine tumors of the gastrointestinal and pancreatobiliary tract, lung, thyroid, and thymoma and confirmed that the SUV of the 68Ga-DOTATATE scan correlated with the score of SSTR2 expression in the respective tissue (40). In a study of lung neuroendocrine tumors, SSTR2 expression in tissue correlated with octreotide scintigraphy in 71% of cases (41).

In our study, age was associated with SSTR2 expression in that patients with TET that expressed SSTR2 in at least 50% of the tumor cells were significantly younger than patients with TET that expressed SSTR2 in 1-49% of tumor cells. While this appears to be a new finding it may, at least in part, be because patients with thymic carcinomas and TNETs were younger than patients with thymoma and SSTR2 was more commonly expressed in thymic carcinomas and TNETs than in thymomas. A multivariate analysis could not be performed given the relative low number of patients.

Our study has several limitations. Although this is one of the largest case series of TET, the overall number of patients who had a 68Ga-DOTATATE PET scan available was relatively small. Furthermore, a comparison group of patients with TET treated with octreotide despite a negative 68Ga-DOTATATE PET scan was not available. Given the low number of cases survival analysis was limited. Also, because of the paucity of TET, patients included in this study were recruited between 1969 and 2021, a relatively long time span during which treatment regimens may have changed. Furthermore, while antigen expression is in general relatively stable in formalin-fixed paraffin-embedded tissue, various fixatives used over time and some degradation are possibilities and could potentially account for lower or lack of expression of SSTR2 in some tumors.

## Conclusions

SSTR2 expression in TET, specifically lymphoepithelial carcinomas, squamous cell carcinomas, atypical carcinoid tumors, and small cell carcinomas may be a biomarker to identify patients who may respond to octreotide therapy. G68-DOTATATE PET scan may be useful to predict expression of SSTR2 in tissue; however, it cannot predict whether SSTR2 is expressed in tumor cells or other cells such as inflammatory cells. Larger, ideally multi-institutional studies are necessary to independently and prospectively validate our results and to correlate SSTR2 expression in TETs and/or 68Ga-DOTATATE PET scans with response to octreotide therapy.

## Data Availability Statement

The original contributions presented in the study are included in the article. Further inquiries can be directed to the corresponding author.

## Ethics Statement

The studies involving human participants were reviewed and approved by Mayo Clinic Rochester Institutional Review Board. Written informed consent for participation was not required for this study in accordance with the national legislation and the institutional requirements.

## Author Contributions

AR, GJ, and AM contributed to the conception and design of the study. AR and SR collected patient information. AR reviewed histologic sections of all cases. GJ reviewed the DOTATATE scans. SJ performed the statistical analysis. AR wrote the first draft of the manuscript. All authors contributed to manuscript revision, read, and approved the submitted version.

## Funding

Mayo Clinic receives funding from Novartis to support research performed by GJ. The funder was not involved in the study design, collection, analysis, interpretation of data, the writing of this article or the decision to submit it for publication.

## Conflict of Interest

AM reports research support from the NIH, DoD, Mark Foundation, Novartis and Verily; remuneration to his institution for participation on advisory boards for AbbVie, Astra Zeneca, BeiGene, BMS, Genentech and Janssen; travel support and payment from Shanghai Roche Pharmaceuticals Ltd.; and is a nonremunerated director of the Mesothelioma Applied Research Foundation. GJ reports that Mayo Clinic receives research funding from Novartis to support his work and that he is on the Advisory Board, Novartis.

The remaining authors declare that the research was conducted in the absence of any commercial or financial relationships that could be construed as a potential conflict of interest.

## Publisher’s Note

All claims expressed in this article are solely those of the authors and do not necessarily represent those of their affiliated organizations, or those of the publisher, the editors and the reviewers. Any product that may be evaluated in this article, or claim that may be made by its manufacturer, is not guaranteed or endorsed by the publisher.

## References

[1] Roden AC Fang W Shen Y Carter BW White DB Jenkins SM. Distribution of Mediastinal Lesions Across Multi-Institutional, International, Radiology Databases. J Thorac Oncol (2020) 15:568–79. doi: 10.1016/j.jtho.2019.12.108 31870881

[2] Roden AC Yi ES Cassivi SD Jenkins SM Garces YI Aubry MC. Clinicopathological Features of Thymic Carcinomas and the Impact of Histopathological Agreement on Prognostical Studies. Eur J Cardiothorac Surg (2013) 43:1131–9. doi: 10.1093/ejcts/ezs529 23086701

[3] Hishida T Nomura S Yano M Asamura H Yamashita M Ohde Y. Long-Term Outcome and Prognostic Factors of Surgically Treated Thymic Carcinoma: Results of 306 Cases From a Japanese Nationwide Database Study. Eur J Cardiothorac Surg (2016) 49:835–41. doi: 10.1093/ejcts/ezv239 26116920

[4] Zhao Y Gu H Fan L Han K Yang J Zhao H. Comparison of Clinical Features and Survival Between Thymic Carcinoma and Thymic Carcinoid Patients. Eur J Cardiothorac Surg (2017) 52:33–8. doi: 10.1093/ejcts/ezx037 28419205

[5] Sakane T Murase T Okuda K Saida K Masaki A Yamada T. A Mutation Analysis of the EGFR Pathway Genes, RAS, EGFR, PIK3CA, AKT1 and BRAF, and TP53 Gene in Thymic Carcinoma and Thymoma Type a/B3. Histopathology (2019) 75:755–66. doi: 10.1111/his.13936 31179560

[6] Bakhos CT Salami AC Kaiser LR Petrov RV Abbas AE. Thymic Neuroendocrine Tumors and Thymic Carcinoma: Demographics, Treatment, and Survival. Innovations (Phila) (2020) 15:468–74. doi: 10.1177/1556984520949287 32938293

[7] Bohnenberger H Strobel P. Recent Advances and Conceptual Changes in the Classification of Neuroendocrine Tumors of the Thymus. Virchows Arch (2021) 478:129–35. doi: 10.1007/s00428-021-03037-1 PMC7965853 33555458

[8] Radovich M Pickering CR Felau I Ha G Zhang H Jo H. The Integrated Genomic Landscape of Thymic Epithelial Tumors. Cancer Cell (2018) 33:244–58. doi: 10.1016/j.ccell.2018.01.003 PMC5994906 29438696

[9] Giaccone G Kim C Thompson J McGuire C Kallakury B Chahine JJ. Pembrolizumab in Patients With Thymic Carcinoma: A Single-Arm, Single-Centre, Phase 2 Study. Lancet Oncol (2018) 19:347–55. doi: 10.1016/S1470-2045(18)30062-7 PMC10683856 29395863

[10] Petrini I Meltzer PS Kim IK Lucchi M Park KS Fontanini G. A Specific Missense Mutation in GTF2I Occurs at High Frequency in Thymic Epithelial Tumors. Nat Genet (2014) 46:844–9. doi: 10.1038/ng.3016 PMC5705185 24974848

[11] Nathany S Tripathi R Mehta A. Gene of the Month: GTF2I. J Clin Pathol (2021) 74:1–4. doi: 10.1136/jclinpath-2020-207013 32907914

[12] Tiseo M Damato A Longo L Barbieri F Bertolini F Stefani A. Analysis of a Panel of Druggable Gene Mutations and of ALK and PD-L1 Expression in a Series of Thymic Epithelial Tumors (TETs). Lung Cancer (2017) 104:24–30. doi: 10.1016/j.lungcan.2016.12.005 28212996

[13] Schirosi L Nannini N Nicoli D Cavazza A Valli R Buti S. Activating C-KIT Mutations in a Subset of Thymic Carcinoma and Response to Different C-KIT Inhibitors. Ann Oncol (2012) 23:2409–14. doi: 10.1093/annonc/mdr626 22357254

[14] Simonelli M Zucali PA Suter MB Lorenzi E Rubino L Fatuzzo G. Targeted Therapy for Thymic Epithelial Tumors: A New Horizon? Review of the Literature and Two Cases Reports. Future Oncol (2015) 11:1223–32. doi: 10.2217/fon.14.318 25832879

[15] Aesif SW Aubry MC Yi ES Kloft-Nelson SM Jenkins SM Spears GM. Loss of P16ink4a Expression and Homozygous CDKN2A Deletion Are Associated With Worse Outcome and Younger Age in Thymic Carcinomas. J Thorac Oncol (2017) 12:860–71. doi: 10.1016/j.jtho.2017.01.028 28179162

[16] Enkner F Pichlhofer B Zaharie AT Krunic M Holper TM Janik S. Molecular Profiling of Thymoma and Thymic Carcinoma: Genetic Differences and Potential Novel Therapeutic Targets. Pathol Oncol Res (2017) 23:551–64. doi: 10.1007/s12253-016-0144-8 PMC5487866 27844328

[17] Katsuya Y Fujita Y Horinouchi H Ohe Y Watanabe S Tsuta K. Immunohistochemical Status of PD-L1 in Thymoma and Thymic Carcinoma. Lung Cancer (2015) 88:154–9. doi: 10.1016/j.lungcan.2015.03.003 25799277

[18] Yokoyama S Miyoshi H Nishi T Hashiguchi T Mitsuoka M Takamori S. Clinicopathologic and Prognostic Implications of Programmed Death Ligand 1 Expression in Thymoma. Ann Thorac Surg (2016) 101:1361–9. doi: 10.1016/j.athoracsur.2015.10.044 26794891

[19] Terra S Mansfield AS Vrana JA Roden AC. Heterogeneity of Programmed Death-Ligand 1 Expression in Thymic Epithelial Tumours Between Initial Specimen and Synchronous or Metachronous Metastases or Recurrences. Histopathology (2019) 74:364–7. doi: 10.1111/his.13750 30182429

[20] Sakane T Murase T Okuda K Takino H Masaki A Oda R. A Comparative Study of PD-L1 Immunohistochemical Assays With Four Reliable Antibodies in Thymic Carcinoma. Oncotarget (2018) 9:6993–7009. doi: 10.18632/oncotarget.24075 29467945 PMC5805531

[21] Lehman JM Hoeksema MD Staub J Qian J Harris B Callison JC. Somatostatin Receptor 2 Signaling Promotes Growth and Tumor Survival in Small-Cell Lung Cancer. Int J Cancer (2019) 144:1104–14. doi: 10.1002/ijc.31771 PMC6448409 30152518

[22] Lehman JM. Amassion PP: Somatostatin Receptor 2 Targeting in Small Cell Lung Carcinoma: Perspectives. Oncotarget (2019) 10:4727–30. doi: 10.18632/oncotarget.27107 PMC6677662 31413814

[23] Lechner M Schartinger VH Steele CD Nei WL Ooft ML Schreiber LM. Somatostatin Receptor 2 Expression in Nasopharyngeal Cancer is Induced by Epstein Barr Virus Infection: Impact on Prognosis, Imaging and Therapy. Nat Commun (2021) 12:117. doi: 10.1038/s41467-020-20308-8 33402692 PMC7785735

[24] Janssen I Chen CC Taieb D Patronas NJ Millo CM Adams KT. 68ga-DOTATATE PET/CT in the Localization of Head and Neck Paragangliomas Compared With Other Functional Imaging Modalities and CT/MRI. J Nucl Med (2016) 57:186–91. doi: 10.2967/jnumed.115.161018 PMC4738157 26564322

[25] Ivanidze J Roytman M Sasson A Skafida M Fahey TJ 3rd Osborne JR. Molecular Imaging and Therapy of Somatostatin Receptor Positive Tumors. Clin Imaging (2019) 56:146–54. doi: 10.1016/j.clinimag.2019.04.006 31121520

[26] Boettcher AN. Somatostatin Receptor Type 2 as an Imaging and Treatment Target for Thyroid Cancer. Radiol Imaging Cancer (2021) 3:e219009. doi: 10.1148/rycan.2021219009 34047669 PMC8183235

[27] Stueven AK Kayser A Wetz C Amthauer H Wree A Tacke F. Somatostatin Analogues in the Treatment of Neuroendocrine Tumors: Past, Present and Future. Int J Mol Sci (2019) 20. doi: 10.3390/ijms20123049 PMC6627451 31234481

[28] Thakur S Daley B Millo C Cochran C Jacobson O Lu H. (177)Lu-DOTA-EB-TATE, a Radiolabeled Analogue of Somatostatin Receptor Type 2, for the Imaging and Treatment of Thyroid Cancer. Clin Cancer Res (2021) 27:1399–409. doi: 10.1158/1078-0432.CCR-20-3453 PMC9531406 33355247

[29] Caplin ME Pavel M Ruszniewski P. Lanreotide in Metastatic Enteropancreatic Neuroendocrine Tumors. N Engl J Med (2014) 371:1556–7. doi: 10.1056/NEJMoa1316158 25317881

[30] Kirzinger L Boy S Marienhagen J Schuierer G Neu R Ried M. Octreotide LAR and Prednisone as Neoadjuvant Treatment in Patients With Primary or Locally Recurrent Unresectable Thymic Tumors: A Phase II Study. PloS One (2016) 11:e0168215. doi: 10.1371/journal.pone.0168215 27992479 PMC5161359

[31] Palmieri G Montella L Martignetti A Muto P Di Vizio D De Chiara A. Somatostatin Analogs and Prednisone in Advanced Refractory Thymic Tumors. Cancer (2002) 94:1414–20. doi: 10.1002/cncr.10374 11920496

[32] Loehrer PJ Sr. Wang W Johnson DH Aisner SC Ettinger DS. Octreotide Alone or With Prednisone in Patients With Advanced Thymoma and Thymic Carcinoma: An Eastern Cooperative Oncology Group Phase II Trial. J Clin Oncol (2004) 22:293–9. doi: 10.1200/JCO.2004.02.047 14722038

[33] Thoracic Tumours. WHO Classification of Tumours 5ed. Lyon, France: International Agency for Research on Cancer (2021).

[34] Amin MB American Joint Committee on Cancer American Cancer Society. AJCC Cancer Staging Manual. Mahul BA Edge SB Gress DM Meyer LR, editors. Chicago IL: American Joint Committee on Cancer, Springer (2017).

[35] Volante M Brizzi MP Faggiano A La Rosa S Rapa I Ferrero A. Somatostatin Receptor Type 2a Immunohistochemistry in Neuroendocrine Tumors: A Proposal of Scoring System Correlated With Somatostatin Receptor Scintigraphy. Mod Pathol (2007) 20:1172–82. doi: 10.1038/modpathol.3800954 17873898

[36] Hu E Levine J. Chemotherapy of Malignant Thymoma. Case Report and Review of the Literature. Cancer (1986) 57:1101–4. doi: 10.1002/1097-0142(19860315)57:6<1101::AID-CNCR2820570606>3.0.CO;2-A 3080220

[37] Viswanathan K Sadow PM. Somatostatin Receptor 2 is Highly Sensitive and Specific for Epstein-Barr Virus-Associated Nasopharyngeal Carcinoma. Hum Pathol (2021) 117:88–100. doi: 10.1016/j.humpath.2021.08.004 34416258 PMC8511208

[38] Shang Q Pang Y Meng T Hao B Chen H. 68ga-DOTATATE PET/CT in Primary Pulmonary Lymphoepithelioma-Like Carcinoma. Clin Nucl Med (2021). doi: 10.1097/RLU.0000000000003857 34319951

[39] Popa O Taban SM Pantea S Plopeanu AD Barna RA Cornianu M. The New WHO Classification of Gastrointestinal Neuroendocrine Tumors and Immunohistochemical Expression of Somatostatin Receptor 2 and 5. Exp Ther Med (2021) 22:1179. doi: 10.3892/etm.2021.10613 34475969 PMC8406677

[40] Miederer M Seidl S Buck A Scheidhauer K Wester HJ Schwaiger M. Correlation of Immunohistopathological Expression of Somatostatin Receptor 2 With Standardised Uptake Values in 68Ga-DOTATOC PET/Ct. Eur J Nucl Med Mol Imaging (2009) 36:48–52. doi: 10.1007/s00259-008-0944-5 18807033

[41] Righi L Volante M Tavaglione V Bille A Daniele L Angusti T. Somatostatin Receptor Tissue Distribution in Lung Neuroendocrine Tumours: A Clinicopathologic and Immunohistochemical Study of 218 ‘Clinically Aggressive’ Cases. Ann Oncol (2010) 21:548–55. doi: 10.1093/annonc/mdp334 19759190

